# Spatial Resolution and Imaging Encoding fMRI Settings for Optimal Cortical and Subcortical Motor Somatotopy in the Human Brain

**DOI:** 10.3389/fnins.2019.00571

**Published:** 2019-06-11

**Authors:** Renaud Marquis, Sandrine Muller, Sara Lorio, Borja Rodriguez-Herreros, Lester Melie-Garcia, Ferath Kherif, Antoine Lutti, Bogdan Draganski

**Affiliations:** ^1^Laboratory for Research in Neuroimaging, LREN, Department of Clinical Neurosciences, Lausanne University Hospital, CHUV, University of Lausanne, Lausanne, Switzerland; ^2^EEG and Epilepsy Unit, Department of Clinical Neuroscience, Faculty of Medicine, Geneva University Hospitals, Geneva, Switzerland; ^3^Lage Lab, Massachusetts General Hospital, Harvard Medical School, Richard B. Simches Research Center, MGH, Boston, MA, United States; ^4^Stanley Center, Broad Institute, Cambridge, MA, United States; ^5^Developmental Neurosciences, UCL Great Ormond Street Institute of Child Health, University College London, London, United Kingdom; ^6^Sensory-Motor Laboratory (SeMoLa), Jules-Gonin Eye Hospital, University of Lausanne, Lausanne, Switzerland; ^7^Max Planck Institute for Human Cognitive and Brain Sciences, Leipzig, Germany

**Keywords:** functional magnetic resonance imaging, segregation, image resolution, BOLD sensitivity, subcortical areas

## Abstract

There is much controversy about the optimal trade-off between blood-oxygen-level-dependent (BOLD) sensitivity and spatial precision in experiments on brain’s topology properties using functional magnetic resonance imaging (fMRI). The sparse empirical evidence and regional specificity of these interactions pose a practical burden for the choice of imaging protocol parameters. Here, we test in a motor somatotopy experiment the impact of fMRI spatial resolution on differentiation between body part representations in cortex and subcortical structures. Motor somatotopy patterns were obtained in a block-design paradigm and visually cued movements of face, upper and lower limbs at 1.5, 2, and 3 mm spatial resolution. The degree of segregation of the body parts’ spatial representations was estimated using a pattern component model. In cortical areas, we observed the same level of segregation between somatotopy maps across all three resolutions. In subcortical areas the degree of effective similarity between spatial representations was significantly impacted by the image resolution. The 1.5 mm 3D EPI and 3 mm 2D EPI protocols led to higher segregation between motor representations compared to the 2 mm 3D EPI protocol. This finding could not be attributed to differential BOLD sensitivity or delineation of functional areas alone and suggests a crucial role of the image encoding scheme – i.e., 2D vs. 3D EPI. Our study contributes to the field by providing empirical evidence about the impact of acquisition protocols for the delineation of somatotopic areas in cortical and sub-cortical brain regions.

## Introduction

Whilst electrophysiological studies provide strong evidence that somatotopy representations in the basal ganglia and thalamus are spatially segregated (Alexander and DeLong, 1985; Nambu, 2011), fMRI studies failed to robustly replicate these findings (Lehéricy et al., 1998; Maillard et al., 2000; Gerardin et al., 2003; Staempfli et al., 2008; Oguri et al., 2013; Zeharia et al., 2015). Up to date, only one single high-resolution imaging study showed clear segregation between somatotopy representations (Staempfli et al., 2008). The controversies in the fMRI literature can be explained by the inherent inter-individual variability of sensorimotor representations, potential differences in fMRI acquisition parameter settings and applied statistical analysis (Picard and Strick, 1996; Beisteiner et al., 2001; Alkadhi et al., 2002). The lack of empirical evidence for the main effects and interactions between these variables calls for the investigation of the effect of fMRI protocol parameters on topology studies of somatotopy representations in both cortical and subcortical regions.

The majority of fMRI studies aiming to differentiate between motor representations of adjacent body parts stress the importance to balance the trade-off between high spatial resolution and the resulting SNR (Kleinschmidt et al., 1997; Hlustik et al., 2001; Kapreli et al., 2007; Meier et al., 2008; Olman et al., 2012). The increase in spatial resolution leads to drop in sensitivity to the BOLD effect that can be partially compensated only for cortical regions using multi-channel receive coils (Triantafyllou et al., 2005, 2011). This is supported by somatotopy studies consistently showing high level of segregation in primary motor cortex (Kapreli et al., 2007; Meier et al., 2008; Zeharia et al., 2012; Cunningham et al., 2013) and SPM (Indovina and Sanes, 2001; Strother et al., 2012), but failing to obtain similar results in deep brain nuclei. The reduction in BOLD sensitivity at higher spatial resolution is particularly pronounced in subcortical areas (Triantafyllou et al., 2005; Lutti et al., 2012), which are inherently associated with low BOLD sensitivity due to their increased iron content and marked distance from the receive elements of the head coil (de Hollander et al., 2017). Furthermore, subcortical regions are more susceptible to physiological noise compared to cortical areas (Hutton et al., 2011; Viviani, 2016; Kasper et al., 2017). In group-level analysis, this marked reduction augments the inter-individual variability that results in poor differentiation between somatotopy areas (Scholz et al., 2000).

There is cumulating empirical evidence about the differential sensitivity of analytical strategies to the regionally specific effect of varying spatial resolution on BOLD sensitivity (Diedrichsen et al., 2011; Molloy et al., 2014; Kirilina et al., 2016). When comparing topological properties previous studies have used the Euclidean distance between centers of gravity or activation maxima (Delmaire et al., 2005; Hashimoto et al., 2013; Besle et al., 2013a), Jaccard or Dice coefficients (Plow et al., 2010; Bracci et al., 2012; Cunningham et al., 2013) and a “selectivity” index that calculates a ratio between BOLD responses (Olman et al., 2012). These analytical techniques provide estimates of distance or similarity between activation clusters that are strongly affected by the amount of noise in the data (Gorgolewski et al., 2010; Stevens et al., 2013). Another limitation of the aforementioned methods is the necessity to set an arbitrary threshold that transforms continuous statistical parametric maps into binary clusters. Voxels with sub-threshold BOLD response are not assigned to any somatotopic cluster and the contribution of voxels that exhibit maximal response is underestimated. More recent study provided an elegant solution, called PCM, that does not require thresholding of activation maps and provides robust inferences despite BOLD sensitivity differences or high percentage of uninformative voxels (Diedrichsen et al., 2011, 2017). PCM has been extensively tested and used in various contexts including motor control (Diedrichsen et al., 2011, 2013a,b, 2017; Ejaz et al., 2015; Diedrichsen and Kriegeskorte, 2017) and can overcome the previous limitations when comparing spatial similarity between motor representations.

In this study, we sought to test the impact of the image resolution – BOLD sensitivity trade-off on spatial differentiation between motor somatotopy representations to provide evidence for optimal fMRI protocol settings for future studies in the field. We hypothesized that image resolution would affect differentially cortical and subcortical areas with more pronounced implications for activations in the deep brain nuclei. Our secondary aim was to test if the spatial differentiation between functional representations can be attributed not only to differential BOLD sensitivity of cortical vs. subcortical areas, but also to the spatial resolution of a given imaging protocol. Motor somatotopy representations were obtained from 1.5, 2, and 3 mm fMRI data acquired during a visually cued motor paradigm. BOLD sensitivity and regression coefficients of the GLM were estimated for each subject and image resolution in cortical and subcortical regions. The levels of segregation of somatotopy maps are calculated using an IoS obtained from the PCM approach (Diedrichsen et al., 2011, 2017). Finally, the estimated segregation levels are compared between fMRI protocols taking in account the corresponding BOLD sensitivity estimates.

## Materials and Methods

### Participants

Sixteen right-handed healthy volunteers (9 females, age range: 18 to 72 years; mean age: 36.6 years, SEM: 4.47 years) were recruited for the study. One individual was discarded from further analysis due to poor data quality. All participants were right-handed (laterality quotient range: 6:20, average = 12.8). Eleven participants indicated preference for right foot and four indicated no preference. The study was approved by the local Ethics committee and participants gave their written informed consent prior to investigation.

### Experimental Paradigm

All volunteers performed the same motor execution task consisting of: (i) unilateral foot movement – flexion and extension of the toes of the right or left foot with the legs resting in flexed position on a platform, (ii) unilateral hand movement – fist opening and closing with the arm kept in a resting position, or (iii) unilateral lower face movement – mouth corners are moved sideward. Right and left body side movements were performed within the same run but in separate blocks. The task was repeated in three separate sessions (runs) corresponding to the different fMRI protocols. Each of the three experimental sessions comprised eighteen blocks of movement repetitions during 16 s, i.e., three for each body part. Blocks of motor activity were interspersed with blocks of rest with the same duration. Before each block, we introduced a motor preparation period consisting of a visual cue with a pictogram of the designated body part accompanied by a countdown of 3 s. Subjects were instructed to move at a pace of 1 Hz indicated by an icon of the corresponding body part displayed at that rate during the active blocks. The rest condition was marked by a fixation cross at the center of the screen and subjects were asked to fixate it. Motor activity blocks were in pseudo-randomized order to prevent bias induced by potential effects of learning, performance and attention. This experiment was realized using Cogent 2000 developed by the Cogent 2000 team at the FIL and the ICN and Cogent Graphics developed by John Romaya at the LON at the Wellcome Department of Imaging Neuroscience. Movement execution was practiced before MRI scanning.

### MRI Acquisition

MRI data was acquired on a Siemens Prisma 3T scanner with a 64-channel head coil. The 1.5 and 2 mm fMRI data were acquired using a 3D encoding scheme (Lutti et al., 2012), the 3 mm data – with a 2D scheme. We used the following acquisition parameters: (i) 1.5 data: TE = 30.9 ms, slice TR = 63 ms, 64 slices, volume TR = 4032 ms, flip angle = 15°, 176 volumes, EPI train length: 47.52 ms (GRAPPA acceleration factor 2 along phase direction), field of view: 192 × 192 in-plane, bandwidth: 1698 Hz/pixel; (ii) 2 mm data: TE = 30 ms, slice TR = 52 ms, 52 slices, volume TR = 2704 ms, flip angle = 15°, 263 volumes, EPI train length: 29.16 ms (GRAPPA acceleration factor 2 along phase direction), field-of-view: 192 × 192 in-plane, bandwidth: 2170 Hz/pixel; (iii) 3 mm data: TE = 30 ms, slice TR = 66 ms, 30 slices, volume TR = 1980 ms, flip angle = 90°, 359 volumes, EPI train length: 35.56 ms, field of view: 192 × 192 in-plane, bandwidth: 2442 Hz/pixel. The fMRI runs with the 3 image resolutions were performed within the same scanning session and their order was pseudo-randomized across subjects. Note that because of slice oversampling, the number of slices and respective slice thickness for each protocol resulted in a different coverage in the head-foot direction during acquisition. Additional slices acquired for 3D EPI protocols were discarded in final images, such that all protocols covered 90 mm in the head-foot direction. The structural MRI data consisted of magnetization transfer (MT) maps (Weiskopf et al., 2013) or T1-weighted (T1w) MPRAGE images (TR = 2000 ms; TI = 920 ms; α = 9°; BW = 250 Hz/pixel; readout in inferior-superior direction; field of view = 256 × 232 mm; 176 slices) at 1 mm resolution. T1w images were used for two subjects whose MT maps quality was impacted by head motion artifacts.

### MRI Data Pre-processing

Data pre-processing and subsequent statistical analysis were performed using the freely available SPM software (SPM12; Wellcome Trust Centre for Neuroimaging^1^) running under Matlab 7.13 (The MathWorks, Inc., Natick, Massachusetts, United States). EPI images were realigned to the subject’s average image across runs and corrected for spatial distortions using the SPM FieldMap toolbox (Hutton, 2002). The parameters of registration to standardized MNI space were calculated on the anatomical image (MT map or T1w image) and the default settings of the “unified segmentation” framework followed by the diffeomorphic registration algorithm DARTEL (Ashburner and Friston, 2005; Ashburner, 2007). The spatial registration parameters were then applied to the functional time-series co-registered to the corresponding individual’s anatomical scan and up-sampled to a uniform 1.5 mm isotropic resolution. Prior to statistical analysis, we applied a spatial smoothing with a Gaussian kernel of 6 mm full-width-at-half-maximum. Because face movements might lead to increased head motion, functional images quality was checked by estimating average scaled variance for each subject and each condition of interest using TSDiffAna SPM extension^2^. Scaled variance was compared across EPI protocols and conditions of interest using a 2-way ANOVA.

### Subject-Level fMRI Modeling

The within-subject statistical analysis was performed using the GLM after convolving the onsets of the active blocks with a canonical hemodynamic response function (Friston et al., 1994, 1995; Worsley and Friston, 1995). We estimated six differential contrasts for each body side and body part separately while using the resting blocks as baseline. Preparation periods and realignment parameters estimated by SPM were included as covariates.

### Group-Level Mass-Univariate Analysis

For the group-level analyses we used three identical flexible-factorial designs corresponding to the three different EPI protocols to include the results from the six differential contrasts as independent levels of a factor. The differential contrasts at the group level tested the positive correlation between movement and BOLD signal changes. Given previously reported motor somatotopy in deep brain nuclei (Staempfli et al., 2008; Nambu, 2011; Zeharia et al., 2015), we expected activity elicited by hand movements to lie primarily in between activity for foot and face. In addition, ordered activity patterns for foot, hand and face movements were expected to predominantly follow a particular direction for each subcortical structure: a dorsal to ventral gradient in the putamen and pallidum, possibly a posterior to anterior gradient in the putamen and a medial to lateral gradient in the pallidum, as well as a lateral to medial gradient in the thalamus. Given the sparse evidence and lack of consistency in motor somatotopy patterns across fMRI studies, we calculated the MNI coordinates of centers of mass and activation maxima in each brain region for each body part and resolution based on group-level results, and tested the congruence of the mapping obtained in our study with the expected somatotopy patterns in two ways. First, we assessed whether the hand was lying in between the two other representations in at least one spatial dimension – along the *X*, *Y*, or *Z* axis. Second, we tested whether the foot and face representations were located at the expected location along the relevant axis, e.g., the foot being more dorsal than the face in the putamen. In addition, we performed a MANOVA on MNI coordinates of centers of mass in *X*, *Y*, and *Z* dimension with body part and resolution as predicting factors across all ROIs. To account for the fact that somatotopic gradient has a different scaling and directionality in each ROI, we applied single value decomposition to MNI coordinates separately for each ROI to project the coordinates of centers of mass in the ROI-specific space defined by principal vectors. Furthermore, we calculated the volume of somatotopic fields for all body parts, ROIs, and resolutions based on group-level mass univariate results to test for a possible link between the activation extend and fMRI resolution as reported in previous studies (Hu and Glover, 2007; van der Zwaag et al., 2009). In order to obtain activation volume in all ROIs for all contrasts and resolutions, statistical maps were thresholded at *p* < 0.05 uncorrected. A 1-way ANOVA evaluated the impact of resolution on activation volume, which were adjusted by dividing volumes by their respective ROI size.

### Pattern Component Modeling

Levels of segregation between functional representations of different body parts were estimated using the PCM approach (Diedrichsen et al., 2011). First, voxel-specific regression coefficients were extracted from the subject-level GLM analysis in the following regions-of-interest (ROIs): M1 [as defined by Van Essen and Drury (1997)], SMA, putamen, pallidum [as defined by the Harvard-Oxford atlas (Frazier et al., 2005; Desikan et al., 2006; Makris et al., 2006; Goldstein et al., 2007)] and thalamus [ventro-lateral and ventral postero-lateral nucleus (as defined by Niemann et al., 2000; Krauth et al., 2010)]. These coefficients were then used as inputs for the PCM, which models the data as a linear combination of pattern components distributed across voxels using a hierarchical Bayesian linear model (Diedrichsen et al., 2011, 2017). In total, we performed at the group level ten PCM analyses – one for each of the five ROI per hemisphere. The PCM is a random-effects model which mainly consists in the following equation:

Y=ZU+E

where *Y* is a *n* by *v* matrix representing the data, *Z* is a *n* by *p* design matrix, *U* is a *p* by *v* matrix representing the pattern components and *E* is a *n* by *v* noise matrix, with *n* being the number of trials, *v* the number of voxels and *p* the number of hypothesized patterns. The errors in *E* are assumed to be independent and identically distributed over trials for single voxels. These patterns are distributions of probabilities over voxels with a voxel-based variance-covariance matrix estimated using an Expectation-Maximization algorithm. Previous reports confirmed the robustness of the estimates against the impact of noise, common activation, and voxel-selection (Diedrichsen et al., 2011).

We provide estimates of similarity between spatial representations (i) across body parts for the different spatial resolutions; (ii) across different spatial resolutions for each body part. Only contralateral representations were considered, such that for each ROI, PCM was performed with a 3 × 3 factorial design (MOVEMENT × RESOLUTION) without constraints on the variance-covariance matrix. We took care that variance and covariance estimates obtained from the PCM were comparable across body parts, resolutions and ROIs by dividing each covariance estimate by the product of its respective variance estimates. Given our aim to compare similarity between representations across conditions rather than likelihood under a particular representational model (Diedrichsen et al., 2011, 2017; Ejaz et al., 2015), we transformed the PCM correlation coefficients using the Fisher r-to-z’ transform (Fisher, 1915, 1921; Sanabria-Díaz et al., 2013) and the absolute value defined our IoS. Given that the interpretation of negative BOLD effects is controversial (Schridde et al., 2008; Schäfer et al., 2012; Zeharia et al., 2012; Huber et al., 2014; Mullinger et al., 2014), we consider the absolute value of Fisher r-to-z’ transform for statistical definition of similarity. IoS represented the absolute value of the inverse hyperbolic tangent of the correlation coefficient *r* between representations:

$$IoS=\left|tanh^{-1}\left(r\right)\right|$$

Low values of IoS indicate high segregation between representations (low *r* values), while high IoS values indicate high similarity (i.e., lack of functional segregation) between representations. Low IoS values indicate low similarity of a given body part across different acquisitions, i.e., the localization is not consistent. PCM provides only one IoS value per pattern component, which resulted in nine IoS values per ROI. After the Fisher’s Z-transform, we obtained the statistical significance of IoS estimates using Z-statistics and FDR correction for multiple comparisons (Benjamini and Hochberg, 1995). In addition, to test for the potential effect of smoothing kernel used for fMRI data pre-processing on IoS values, functional time-series were analyzed using: (i) spatial Gaussian smoothing kernel proportional to the particular image resolution; (ii) left without spatial smoothing. We then repeated the GLM, PCM and IoS estimation on the resulting outputs of these two alternative strategies.

### BOLD Sensitivity Analyses

As measures of BOLD sensitivity, we provide average t-scores per ROI within the 5% most significant voxels and estimates of the tSNR at the voxel and ROI levels. For the calculation of tSNR, we removed the effects of the original time-series at the subject level (Lutti et al., 2012) using standard pre-processing with rigid transformation within and between sessions, image distortion correction and spatial registration to MNI space. We present Pearson’s and Spearman’s correlation coefficients between the IoS and tSNR values across all ROIs and image resolutions. Both Pearson’s and Spearman’s correlation coefficients were used in order to characterize the nature of the presumed relationship between tSNR and IoS and provide linear as well as non-linear measures. In addition, we estimated the combined effects of tSNR and image resolution (coded as factor with 3 levels) on IoS using analysis-of-covariance (ANCOVA) in R 3.3.1 and the R package car 2.1-3 (Fox and Weisberg, 2011; R Core Team, 2012). The contribution of tSNR to the effect of image resolution on IoS values was assessed using model comparison between the ANCOVA model and a simpler regression model including only tSNR as predictor. We performed planned *post hoc* comparisons using the R package phia 0.2-1. For the correlation coefficients calculation that entered the ANCOVA model we averaged IoS values across pairs of contrasts and tSNR values across subjects. We also performed the same analyses with tSNR estimates weighted by dividing the values by the squared root of the corresponding volume TR (*tSNR*/$\sqrt{TRvolume}$; Poser et al., 2010), the number of volumes (*tSNR*/$\sqrt{N}$; Smith et al., 2013), and tSNR values scaled by a factor that includes the number of volumes and accounts for autocorrelation in the data (*tSNR_s_*; Todd et al., 2016). In addition, because head motion during acquisition can affect tSNR estimates, we calculated average and maximum head motion, number of head movements above 0.5 mm, as well as rotations (Van Dijk et al., 2012) and performed 1-way ANOVAs on each metric with resolution as predicting factor.

## Results

### Mass-Univariate Analyses

**FIGURE 1:**
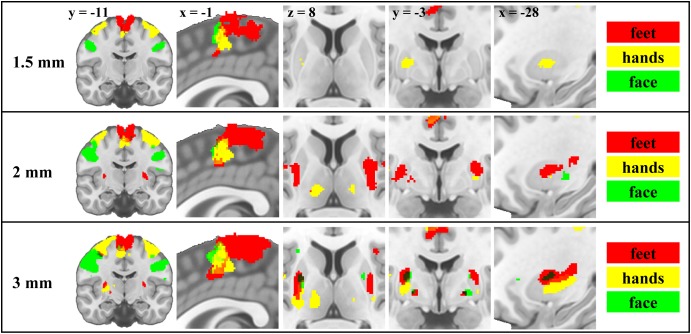
Motor somatotopy patterns across resolutions and brain regions projected on canonical anatomical image in standard space. Group results obtained using the flexible factorial design showing the binarised statistical parametric maps (*t*-values) thresholded at α = 0.05 (corrected for multiple comparisons, family-wise error rate) for each resolution. Left and right body movements are merged (red – feet; yellow – hands; green – face).

The group level analysis demonstrated somatotopy patterns in cortical and subcortical areas (Figure 1 and Supplementary Figures S1, S6–S9). We report activations in primary motor cortical areas, thalamus, putamen and pallidum (whole brain results table, MNI coordinates of maxima, centers of mass and activation volume available as Supplementary Material). Consistent with previous motor somatotopy studies, foot, hand and face representations were located along a dorsal to ventral and medial to lateral gradient in M1 and along a posterior to anterior gradient in the SMA. In the putamen, foot activity was more anterior and medial than the face and more ventral than the hand, but locations of face and hand activity were consistent with the study of Staempfli et al. (2008). In the pallidum, the foot was more ventral than the face. In the thalamus, face activity was more anterior, ventral and medial compared to hand, and more posterior, dorsal and lateral compared to foot. However, in deep brain nuclei, coordinates of centers of mass and activation maxima were only partially congruent with the expected somatotopy. Centers of mass of hand representations were in between foot and face along at least one dimension in 55.6% of subcortical ROIs across all resolutions (50% for 1.5 mm, 83.3% for 2 mm, and 33.3% for 3 mm). For activation maxima, this spatial ordering was lower (38.9%) on average (38.9%) but higher (66.7%) for 3 mm resolution (16.7% for 1.5 mm, and 33.3% for 2 mm). This ordering was nevertheless always confirmed in the putamen and pallidum except in the right putamen for 1.5 mm and in the pallidum for 3 mm. In the thalamus, only 2 mm data were associated with this specific spatial ordering of centers of mass. Centers of mass of foot representation were more dorsal than the face only in the left pallidum for 3 mm and left putamen for 2 mm resolution, and foot was systematically more medial than face activity in the thalamus. Activation maxima were more dorsal for foot than for face only in the putamen using 3 mm data.

The 2-way MANOVA results revealed a significant effect of body part (Pillai’s trace = 0.39; *p* < 0.001) on centers of mass coordinates in principal axes across ROIs, but no effect of resolution (Pillai’s trace = 0.02; *p* = 0.921) and no interaction between resolution and body part (Pillai’s trace = 0.10; *p* = 0.783). All ANOVAs performed on each dimension separately revealed a significant effect of body part [first principal axis: *F*(2,81) = 13.86, *p* < 0.001; second principal axis: *F*(2,81) = 9.71, *p* < 0.001; third principal axis: *F*(2,81) = 3.72, *p* = 0.029], no resolution effect and no interaction. *Post hoc* tests using Bonferroni-Holm correction for multiple comparisons showed significant differences between face and foot [*F*(1,81) = 15.85; *p* < 0.001] and between foot and hand [*F*(1,81) = 24.75; *p* < 0.001] for the first principal axis, between the same pairs of body parts for the second principal axis [*F*(1,81) = 10.32; *p* = 0.004 and *F*(1,81) = 17.80; *p* < 0.001, respectively], and between face and foot [*F*(1,81) = 7.19; *p* = 0.027] for the third principal axis.

There was a significant effect of resolution on activation volume [*F*(2,87) = 6.52; *p* = 0.002] as shown by the 1-way ANOVA (*R*^2^ = 0.13; adjusted *R*^2^ = 0.11). Average activation volume adjusted for ROI size was 0.40 for 1.5 mm, 0.60 for 2 mm, and 0.56 for 3 mm data. *Post hoc* tests using Bonferroni-Holm correction showed a difference between 1.5 mm and 2 mm [*F*(1,87) = 11.78; *p* = 0.003] and between 1.5 mm and 3 mm [*F*(1,87) = 7.23; *p* = 0.017].

The 2-way ANOVA performed on scaled signal variance revealed a significant model fit [*F*(17,252) = 4.19; *p* < 0.001; *R*^2^ = 0.22; adjusted *R*^2^ = 0.17] and an effect of EPI protocol on image quality [*F*(2,252) = 34.27; *p* < 0.001; Supplementary Figure S5]. Neither the effect of movement type [*F*(5,252) = 0.29; *p* = 0.92], nor the interaction between movement type and EPI protocol were significant [*F*(2,252) = 0.13; *p* > 0.99].

### Indices of Similarity

**FIGURE 2:**
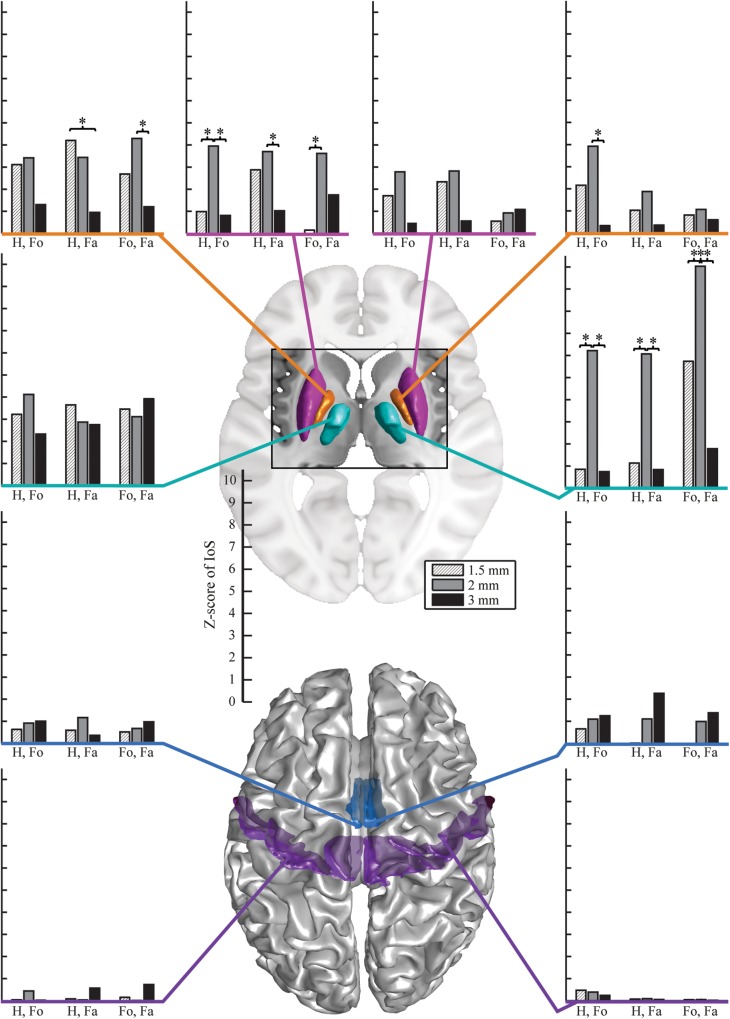
Z-scores of IoS for hand against foot (Ha. vs. Fo.), hand against face (Ha. vs. Fa.) and foot against face (Fo. vs. Fa.) per resolution and ROI, projected on canonical anatomical image in standard space. Bar plots on the left are for left ROIs, bar plots on the right are for right ROIs. Surface renderings of the putamen (magenta), pallidum (orange), motor nuclei of the thalamus (cyan), SMA (mid-tone blue) and M1 (violet). EPI protocols denoted by hatched light gray – 1.5 mm; mid-tone gray – 2 mm, black – 3 mm). Stars indicate significantly different Z-scores (*p* < 0.05, FDR-corrected).

**FIGURE 3:**
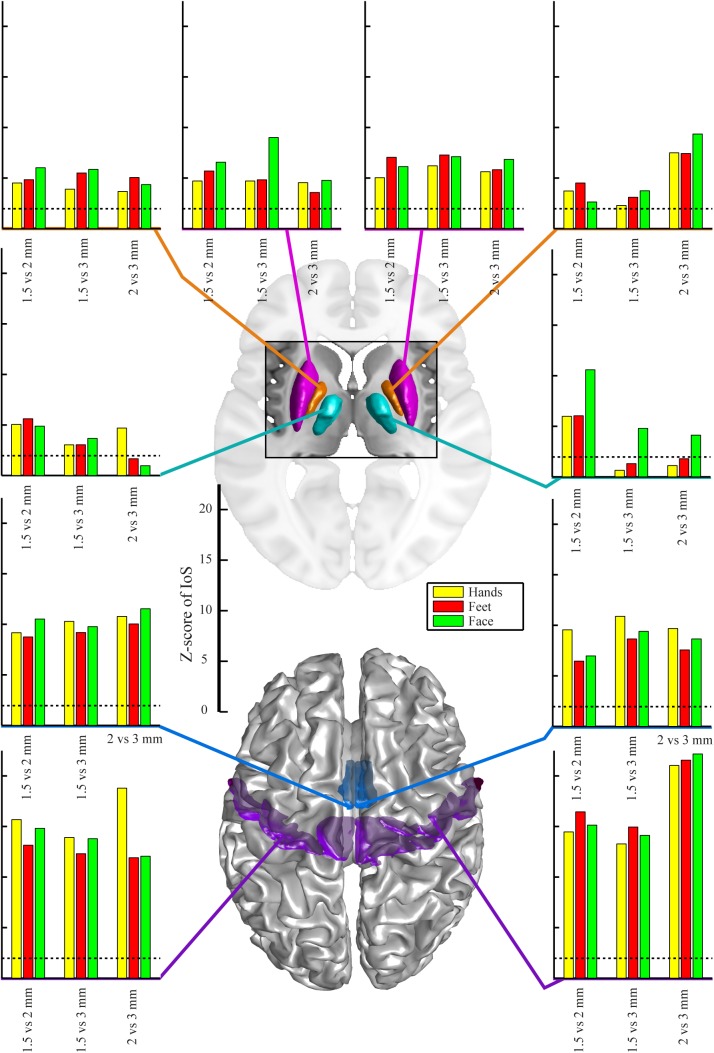
Z-scores of IoS pair-wise comparison for 1.5 mm, 2 mm and 3 mm resolution for different body parts – hands, feet, face and regions-of-interest, projected on canonical anatomical image in standard space. Regions-of-interest in putamen (magenta), pallidum (orange), motor nuclei of the thalamus (cyan), SMA (mid-tone blue) and M1 (violet). Dotted lines indicate significance of correlation (*p* < 0.05 uncorrected for multiple comparisons, bilateral test).

There were no significant differences between EPI protocols in cortical ROIs when comparing the IoS Z-scores across pairs of movements for each resolution and ROIs (Figure 2). We observed differences in subcortical ROIs where 3 mm provided lower IoS values compared to 1.5 mm in the left pallidum (*p* = 0.01) and right thalamus (*p* = 0.001). IoS values were significantly lower for 1.5 mm as compared to 2 mm EPI in the left putamen (*p* = 0.02) and right thalamus (*p* < 0.001). IoS values were lower for 3 mm compared to 2 mm EPI in the left putamen (*p* = 0.01), left pallidum (*p* = 0.01) and the right thalamus (*p* < 0.001). All z-scores of motor representations between EPI protocols were significant (*p* < 0.05, FDR-corrected), except for 3 mm EPI against other protocols in the thalamus, showing that motor representations were robustly mapped and were associated with a consistent localization (Figure 3). Similar results were obtained when different smoothing strategies were used (Supplementary Figure S2).

### Contribution of BOLD Sensitivity to Pattern Similarity

**FIGURE 4:**
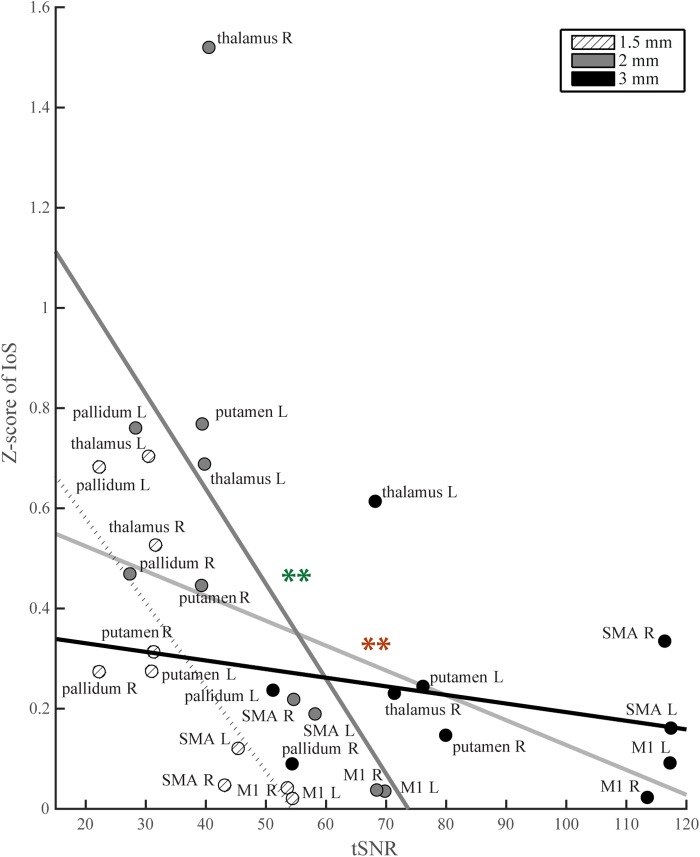
IoS values per ROI as a function of tSNR. Light gray line: regression line across all image resolutions, significance denoted with red stars; thick black line: regression line for 3 mm data; thick dark gray: regression line for 2 mm data, significance denoted with green stars; dotted line: regression line for 1.5 mm data.

For each ROI, t-scores were higher with lower image resolution (Supplementary Figure S3). Similarly, whole brain tSNR maps showed the highest values for 3 mm and the lowest for 1.5 mm EPI (Supplementary Figure S4). We found a negative correlation between tSNR and IoS values (*r* = -0.43; *p* = 0.017; ρ = -0.58; *p* < 0.001) (Figure 4). 3 mm EPI was generally associated with high tSNR and low IoS values. IoS was high and tSNR low for 2 mm resolution, and both tSNR and IoS values were low for 1.5 mm EPI (Figure 4). The ANCOVA showed significant model fit [*F*(5, 24) = 4.98; *p* = 0.003; *R*^2^ = 0.51; adjusted *R*^2^ = 0.41], providing better fitting than the regression model considering only tSNR (*R*^2^ = 0.19; adjusted *R*^2^ = 0.16) as confirmed by model comparison [*F*(4,24) = 3.93; *p* = 0.014]. We found a significant effect of tSNR [*F*(1,24) = 8.24; *p* = 0.008] on IoS estimates but only a trend for image resolution [*F*(2,24) = 3.17; *p* = 0.06]. In addition, there was a significant interaction between tSNR and image resolution [*F*(2,24) = 4.7; *p* = 0.019]. The effect of tSNR on IoS was significant for 2 mm [*F*(1,24) = 10.98; *p* = 0.009] but not for 3 mm [*F*(1,24) = 4.67; *p* = 0.061] and 1.5 mm [*F*(1,24) = 0.06; *p* = 0.817]. However, none of the contrasts between image resolution showed a significant differential effect of tSNR on IoS across image resolutions (*p* > 0.2). Results of correlation tests and ANCOVA using weighted BOLD sensitivity metrics were comparable to those using unweighted tSNR and are reported in Table 1. None of the 1-way ANOVAs showed any significant effect of resolution on any head motion metric [average head motion: mean = 0.39, 0.37 and 0.38 mm for 1.5, 2, and 3 mm, respectively, *F*(2,42) = 0.08, *p* = 0.923; maximum head motion: mean = 0.91, 0.87, and 1.03 mm, *F*(2,42) = 0.66, *p* = 0.523; number of head movements: mean = 48.53, 61.87, and 92.27, *F*(2,42) = 2.56, *p* = 0.090; rotations: mean = 45 × 10^-4^, 44 × 10^-4^ and 49 × 10^-4^ degrees, *F*(2,42) = 0.15, *p* = 0.863].

**Table 1:** Results of ANCOVA and correlation analysis of BOLD sensitivity metrics using F-statistics.

		*tSNR*	*tSNR_s_*	$\frac{tSNR}{\sqrt{TRvolume}}$	$\frac{tSNR}{\sqrt{N}}$
*BOLD sensitivity*	F	8.24	7.17	6.51	6.5
	p	0.008**	0.013*	0.018*	0.018*
*Resolution*	F	3.17	3.02	3.45	3.45
	p	0.06	0.068	0.048*	0.048*
*BOLD sensitivity x Resolution*	F	4.7	5.22	5.56	5.56
	p	0.019*	0.013*	0.01*	0.01*
*Model fitting*	F	4.98	4.97	4.98	4.98
	p	0.003**	0.003**	0.003**	0.003**
*R^2^*		0.51	0.51	0.51	0.51
*Adjusted R^2^*		0.41	0.41	0.41	0.41
*r (Pearson)*		-0.43	-0.41	-0.38	-0.38
*p*		0.017*	0.023*	0.041*	0.042*
*ρ (Spearman)*		-0.58	-0.56	-0.48	-0.48
*p*		<0.001***	0.002**	0.009**	0.009**


^∗^p < 0.05, ^∗∗^p < 0.01, and ^∗∗∗^p < 0.001.

## Discussion

In this study, we demonstrate the impact of fMRI protocol settings on neural activity patterns in cortical and subcortical brain regions. We estimated the main effect and interactions of spatial resolution and image encoding on the ability to separate somatotopy representations in cortical and subcortical areas whilst acknowledging the regionally specific differential BOLD sensitivity. Higher image resolution did not improve the segregation between body part representations in the cortex. Conversely, motor somatotopy patterns in deep brain nuclei were better segregated at both high and low, but not intermediate spatial resolution, suggesting a crucial role for image encoding scheme.

Given that previous studies demonstrated the impact of fMRI data pre-processing (Geissler et al., 2005), experimental design(Besle et al., 2013b) and statistical analysis (Dechent and Frahm, 2003) on topology properties of neural activity, we kept these parameters unchanged across spatial resolutions. Although body part representations did not strictly follow the spatial ordering and location of motor somatotopy patterns as expected from electrophysiological recordings in primates in a systematic fashion, MANOVA results showed that the location of centers of mass across ROIs was determined by the body part moved rather than the fMRI protocol. Moreover, we systematically observed high similarity values between representations of the same body part across different resolutions, except in the thalamus. These results suggest that fMRI resolution did not change the location of representations but their extent.

In cortical areas we observed the same level of segregation between somatotopy maps across all image resolutions, which is consistent with the reduced impact of high-resolution on BOLD sensitivity in these regions (Triantafyllou et al., 2011). Conversely, high image resolution might prove advantageous to delineate smaller somatotopy representations such as within-limb or finger somatotopy (Kleinschmidt et al., 1997; Dechent and Frahm, 2003; Plow et al., 2010). In subcortical areas, we found significant discrepancies in delineation between the investigated image resolutions. In particular, the 2 mm 3D EPI data yielded the smallest levels of segregation between motor representations. 3 mm 2D led to higher levels of segregation than 2 mm 3D EPI and to even occasionally outperform 1.5 mm 3D EPI. These results rule out a linear effect of improved delineation with higher resolution data. The better delineation at 3 mm spatial resolution points toward a predominant contribution of BOLD sensitivity in driving these effects – the latter being about twice as large for 3 mm compared to 1.5 mm data. It is of note that IoS estimates in motor thalamic ROIs were especially high. 2 mm EPI was associated with extreme values in the right but not in the left thalamus despite symmetric values of tSNR and t-scores in those regions. Due to higher levels of noise in the region the thalamus results should be cautiously interpreted awaiting confirmation by cross-validation (Diedrichsen et al., 2013a).

Correlation analysis confirmed that BOLD sensitivity has a significant effect on estimates of segregation between neural representations. The 1.5 mm protocol delivered better segregation than the 2 mm one, which suggests that BOLD sensitivity alone does not explain segregation estimates, and that accounting for image resolution better explains variability in somatotopy delineation. Similarly, the effect of BOLD sensitivity on segregation estimates vary as a function of the fMRI protocols. It is likely that the encoding scheme used – i.e., 2D vs. 3D, is a determining factor of the obtained results. Studies point toward larger activation cluster extent of 3D compared to 2D EPI schemes (Hu and Glover, 2007; van der Zwaag et al., 2009). This effect was attributed to physiological noise (Lutti et al., 2012; Jorge et al., 2013) mostly affecting sub-cortical regions (Hutton et al., 2011), which is consistent with our observations. In our study, 1.5 mm protocol was associated with smaller volumes of activation but 2 mm was associated with the largest volumes. However, as shown by the ANOVA results, head motion alone cannot explain BOLD sensitivity differences and is hence unlikely related to the different IoS values observed. The findings of superior segregation at 1.5 mm compared with 2 mm 3D EPI are interpreted in the context of improved delineation of motor areas or reduced contribution of physiological noise in high resolution data (Triantafyllou et al., 2011). Thus, our decision not to acquire physiological data might have a significant impact on the subcortical read-outs given that cardiac and respiratory artifacts are accentuated in these regions and that 3D readouts are more affected by physiological noise. However, the regional segregation of 1.5 mm data remained lower than the 3 mm data, which motivates the combined study of physiological correction and 1.5 mm 2D EPI in future studies.

Similarly, systematic differences in brain coverage volume can lead to differential BOLD in 2D and 3D EPI acquisition schemes (Poser et al., 2010), thus explaining why 1.5 and 3 mm EPI outperform 2 mm resolution data. Besides image resolution, another important factor influencing BOLD sensitivity is the choice of TE, especially at ultra-high field (de Hollander et al., 2017), which motivated our decision to keep it identical across protocols. We also decided for the same fixed duration of data acquisition to reflect in a more ecological way the impact of different number of acquired volumes with varying image resolutions on BOLD sensitivity. One could have kept the number of volumes acquired constant across fMRI protocols that will lead to substantial increase for the high-resolution fMRI acquisitions. This would not only allow for calculating robustness estimates at variable number of data points, but also for fMRI protocol comparisons adjusted for BOLD sensitivity differences across image resolutions.

Another limitation of our study is the lack of tight control of behavioral performance, which can lead to drop in robustness of somatotopy patterns due to co-occurrence of limb joint movements (Luft et al., 2002). Our findings support the notion of interaction between spatial resolution and image encoding scheme on topological properties of neural activity related to motor action. Keeping in mind that we use a 2D acquisition for our 3 mm fMRI protocol, we assume that only the inferences based on 1.5 and 2 mm data can be interpreted in straightforward way. Along the same lines, one potential extension of the current study is to investigate somatotopy related topology properties with multiband fMRI acquisition protocols, which are becoming increasingly common.

In summary, we provide empirical evidence for a differential impact of fMRI protocols spatial resolution and encoding scheme – 2D vs. 3D, on cortical and subcortical motor somatotopy resulting from the complex interaction of spatial heterogeneity of factors related to BOLD sensitivity. The presented analytical strategy and the use of a dedicated index-of-segregation could help future studies to make informed decisions of optimal fMRI protocol setting when studying somatotopy patterns in the human brain. Based on our findings, we recommend a careful selection of fMRI protocol settings depending on the focus of the study – particularly, segregating between emphasis on cortical or subcortical regions. Given the specific tissue properties of deep brain nuclei featuring high iron content of basal ganglia that increase with age, we suggest either using 3 mm 2D or 1.5 mm 3D acquisition. Although not supported by our experimental setting lacking physiological parameter recordings, we recommend physiological noise correction for future studies on cortical and subcortical motor somatotopy.

## Ethics Statement

The study was carried out in accordance with the recommendations of Commission cantonale d’éthique de la recherche sur l’être humain with written informed consent from all subjects. All subjects gave written informed consent in accordance with the Declaration of Helsinki. The protocol was approved by the Commission cantonale d’éthique de la recherche sur l’être humain.

## Author Contributions

RM, AL, FK, and BD contributed to the design and implementation of the research. AL designed the EPI protocols. RM carried out the experiment. RM analyzed the data with support from SM, SL, and LM-G. RM wrote the manuscript with support from SM, SL, BR-H, LM-G, FK, AL, and BD. AL and FK helped to supervise the study. BD supervised the project. All authors discussed the methods and results of this study and contributed to the final work.

## Conflict of Interest Statement

The authors declare that the research was conducted in the absence of any commercial or financial relationships that could be construed as a potential conflict of interest.

## Footnotes

1 http://www.fil.ion.ucl.ac.uk/spm/

2 https://www.fil.ion.ucl.ac.uk/spm/ext

## Supplementary Material

FIGURE S1 Motor somatotopy across resolutions and brain regions projected on canonical anatomical image in standard space. Binarised statistical parametric maps (*t*-values) thresholded at α = 0.001 uncorrected for multiple comparisons, minimal cluster extent of 10 voxels for different spatial resolutions.

FIGURE S2 Effect of different spatial smoothing strategies on PCM results. Z-scores of pair-wise comparisons of IoS across ROIs and spatial resolution. Top: 6 mm FWHM Gaussian kernel, middle: Gaussian kernel with full-width-at-half-maximum proportional to EPI protocol spatial resolution – 4.5, 6, and 9 mm for 1.5, 2, and 3 mm EPI, bottom: no spatial smoothing.

FIGURE S3 Average somatotopy t-scores across regions-of-interest and spatial resolution. Bars representing mean individuals’ T-scores with error bars indicated standard error in the 5% most significant voxels within the ROI.

FIGURE S4 Voxel-wise temporal signal-to-noise ratio (tSNR) maps averaged across individuals.

FIGURE S5 Average scaled signal variance of somatotopy maps across spatial resolutions.

FIGURE S6 Surface rendering of motor somatotopy patterns in subcortical regions as a function of resolution and statistical threshold projected on canonical anatomical image in standard space. Group results from flexible factorial design represented as binarised statistical parametric maps after *F*-test across subcortical regions-of-interest, spatial resolution and statistical threshold (*p* < 0.001, 0.01, and 0.05, uncorrected for multiple comparisons, minimal cluster extent of 10 voxels). Filled circles indicate coordinate with maximal *F*-value for each contrast (red, feet; yellow, hands; green, face). White arrows in the top left image indicate directions of anatomical landmarks (S, superior; L, left; A, anterior).

FIGURE S7 2D and 3D representations of motor somatotopy at 1.5 mm resolution projected on canonical anatomical image in standard space. Non-thresholded statistical maps – F-contrasts for feet (red), hands (yellow) and face (green) at α = 0.01 uncorrected with a minimal cluster extent of 10 voxels. White arrows – directions for anatomical landmarks (S, superior; L, left; A, anterior).

FIGURE S8 2D projections of centers of mass coordinates for foot (red), hand (yellow) and face (green) movements at 1.5 (upward triangle), 2 (diamond) and 3 (downward triangle) mm resolution along X (a, b, g, h, m, and n), Y (c, d, i, j, o, and p) and Z (e, f, k, l, q, and r) planes in the putamen (upper row), pallidum (middle row), and thalamus (bottom row). Circles represent the average coordinates of centers of mass across resolution.

FIGURE S9 2D projections of activation maxima coordinates for foot (red), hand (yellow) and face (green) movements at 1.5 (upward triangle), 2 (diamond) and 3 (downward triangle) mm resolution along X (a, b, g, h, m, and n), Y (c, d, i, j, o, and p) and Z (e, f, k, l, q, and r) planes in the putamen (upper row), pallidum (middle row), and thalamus (bottom row). Circles represent the average coordinates of activation maxima across resolution.

TABLE S1 Whole brain group results table (spreadsheet “SPM_results”), reporting *F*- and *t*-values for the three separate design matrices for each resolution (thresholded at *p* < 0.001 uncorrected and cluster extent *k* > = 10); MNI coordinates of maxima (spreadsheet “Maxima”) for each contrast, ROI and resolution; activation volume (mm3) and MNI coordinates of centers of mass (spreadsheet “Activated_volume_and_COM”) for each contrast, ROI and resolution at different thresholds (*p* < 0.001, *p* < 0.01, and *p* < 0.05, uncorrected).

## Abbreviations

ANCOVA: analysis of covariance
ANOVA: analysis of variance
BOLD: blood-oxygen-level-dependent
DARTEL: diffeomorphic anatomical registration using exponentiated lie algebra
EPI: echo-planar imaging
FDR: false discovery rate
fMRI: functional magnetic resonance imaging
GLM: general linear model
IoS: index of similarity
M1: primary motor cortex
MNI: Montreal Neurological Institute
MRI: magnetic resonance imaging
PCM: pattern component model
ROI: region of interest
SEM: standard error of the mean
SMA: supplementary motor area
SNR: signal-to-noise ratio
SPM: statistical parametric mapping
TE: echo time
TR: repetition time
tSNR: temporal SNR
