# Supplementary figures and images for: Spatial Resolution and Imaging Encoding fMRI Settings for Optimal Cortical and Subcortical Motor Somatotopy in the Human Brain
Source: Front Neurosci. 2019 Jun 11;13:571. doi: 10.3389/fnins.2019.00571 (PMC6579882; doi:10.3389/fnins.2019.00571)

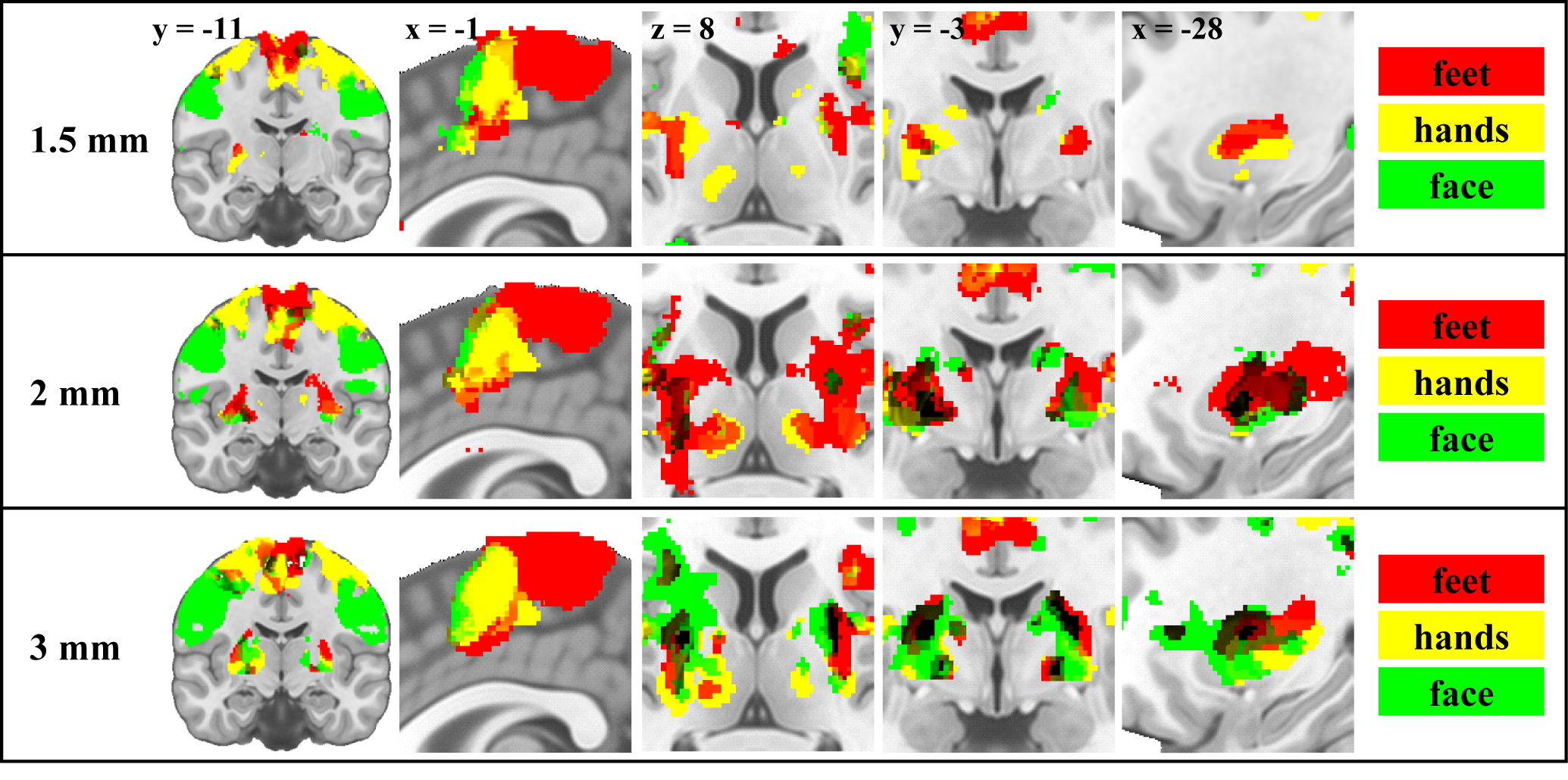

Supplement: FIGURE S1 — Motor somatotopy across resolutions and brain regions projected on canonical anatomical image in standard space. Binarised statistical parametric maps (t-values) thresholded at α = 0.001 uncorrected for multiple comparisons, minimal cluster extent of 10 voxels for different spatial resolutions. [file Image_1.TIF]

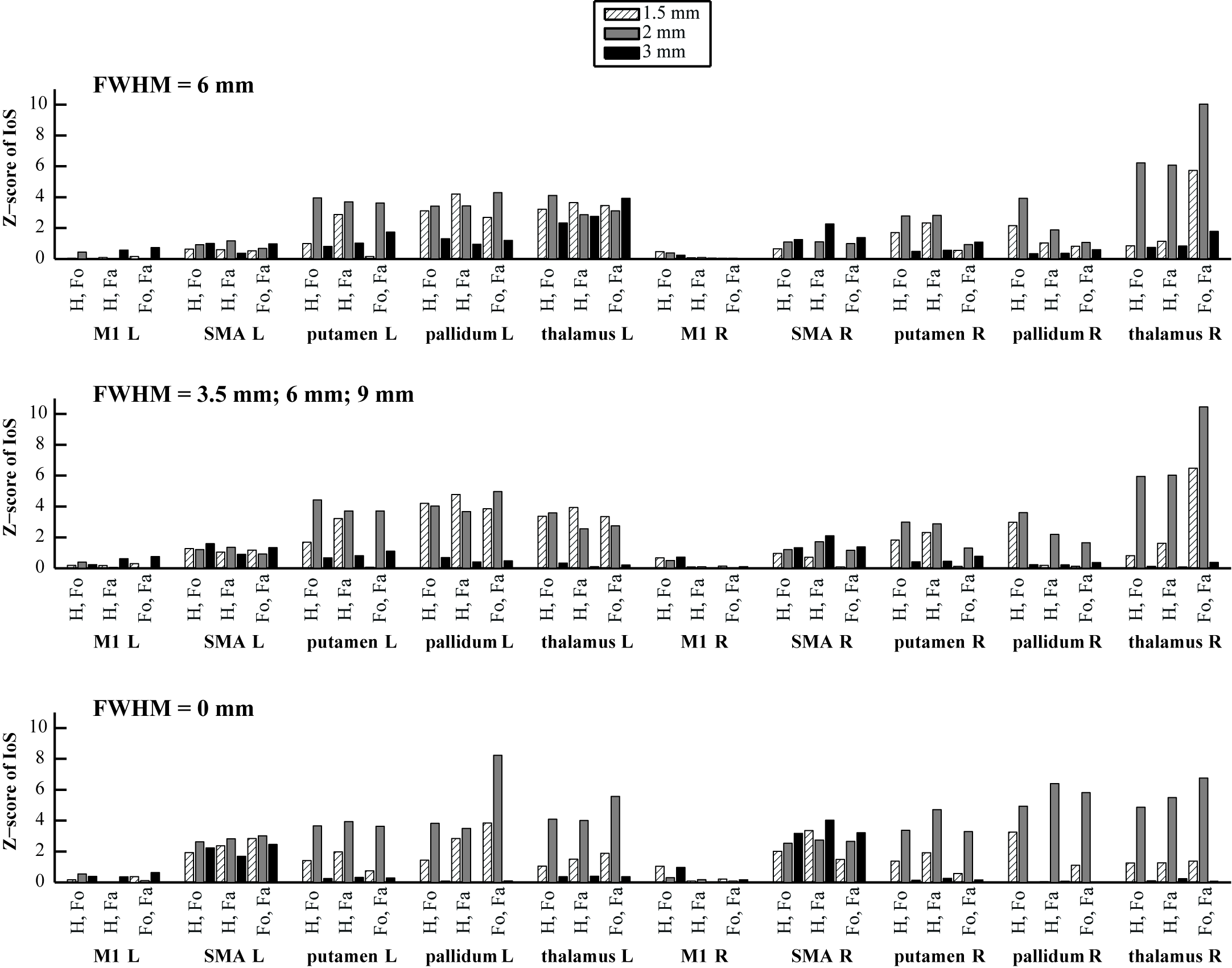

Supplement: FIGURE S2 — Effect of different spatial smoothing strategies on PCM results. Z-scores of pair-wise comparisons of IoS across ROIs and spatial resolution. Top: 6 mm FWHM Gaussian kernel, middle: Gaussian kernel with full-width-at-half-maximum proportional to EPI protocol spatial resolution – 4.5, 6, and 9 mm for 1.5, 2, and 3 mm EPI, bottom: no spatial smoothing. [file Image_2.TIF]

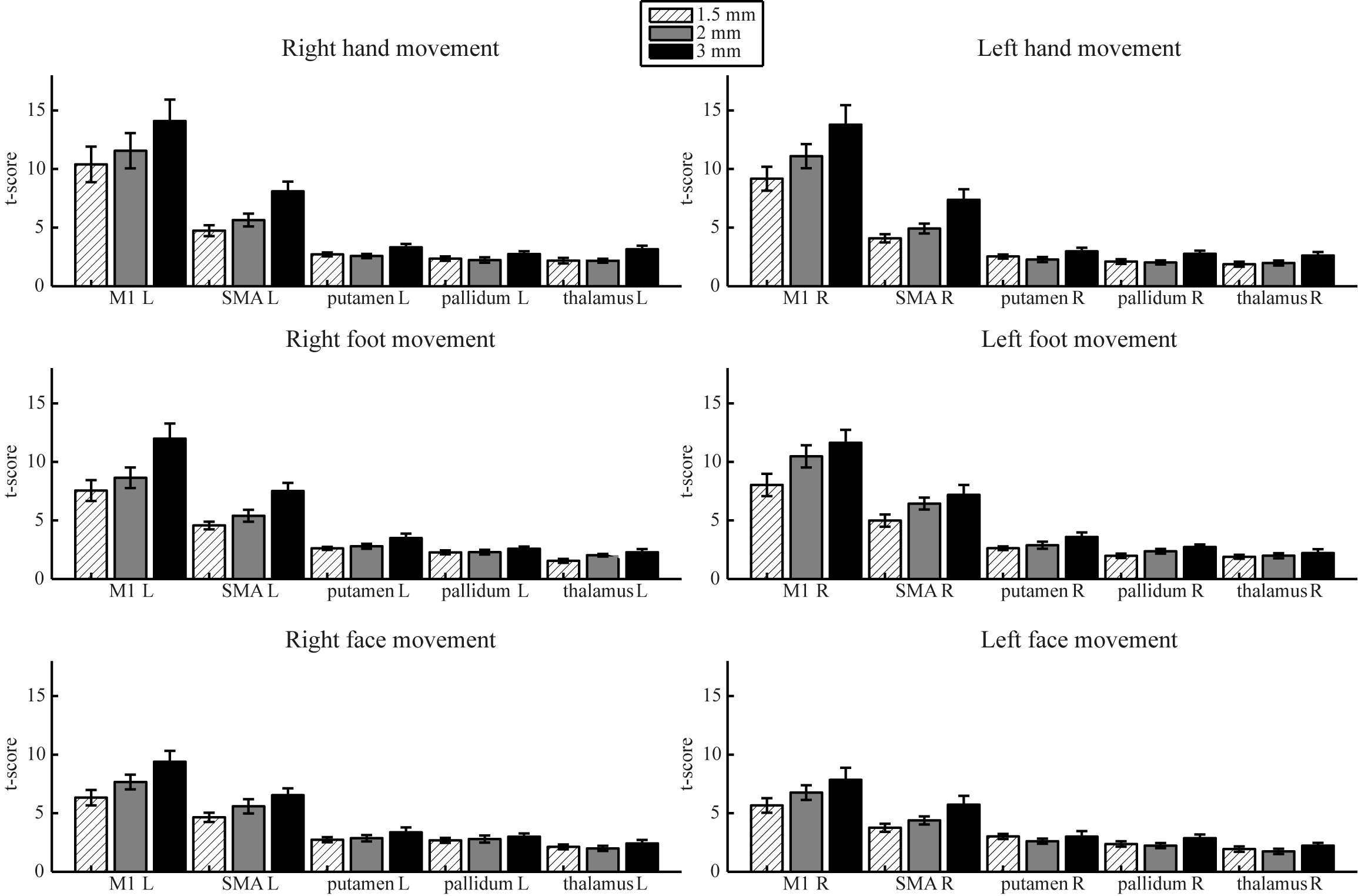

Supplement: FIGURE S3 — Average somatotopy t-scores across regions-of-interest and spatial resolution. Bars representing mean individuals’ T-scores with error bars indicated standard error in the 5% most significant voxels within the ROI. [file Image_3.TIF]

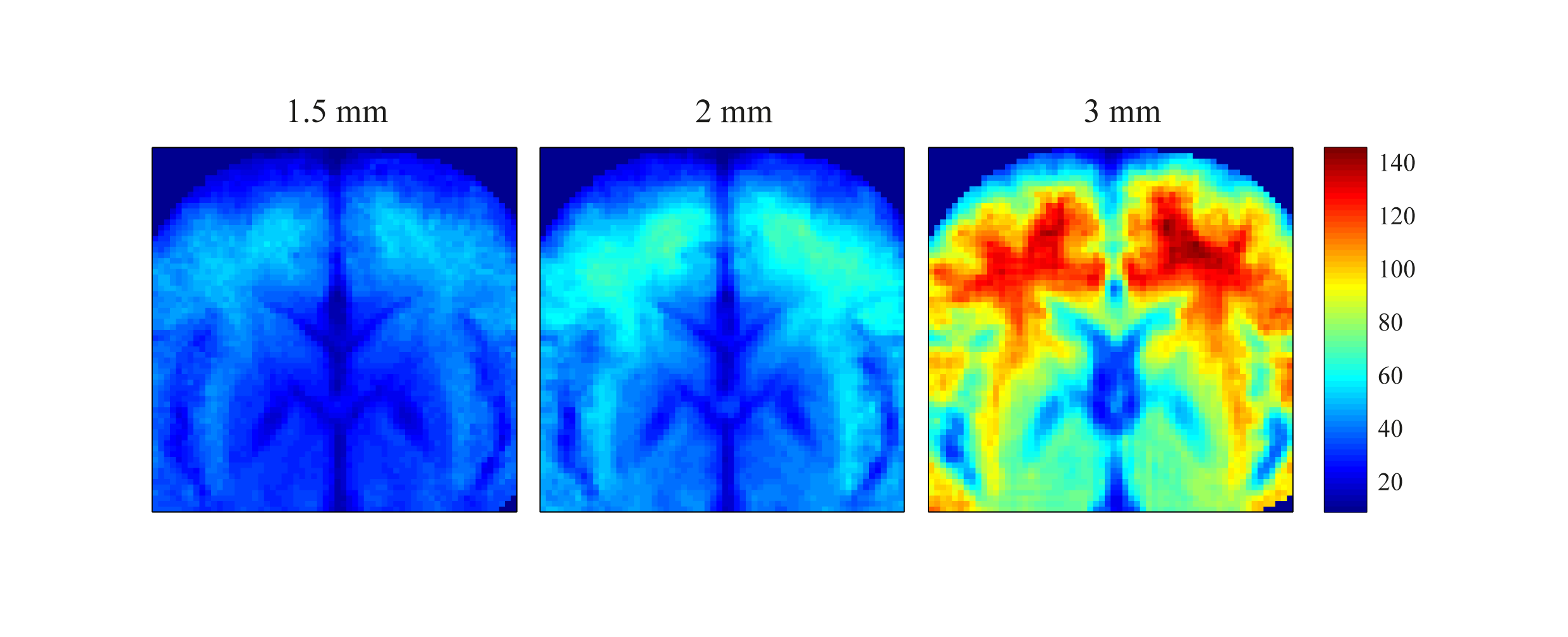

Supplement: FIGURE S4 — Voxel-wise temporal signal-to-noise ratio (tSNR) maps averaged across individuals. [file Image_4.TIF]

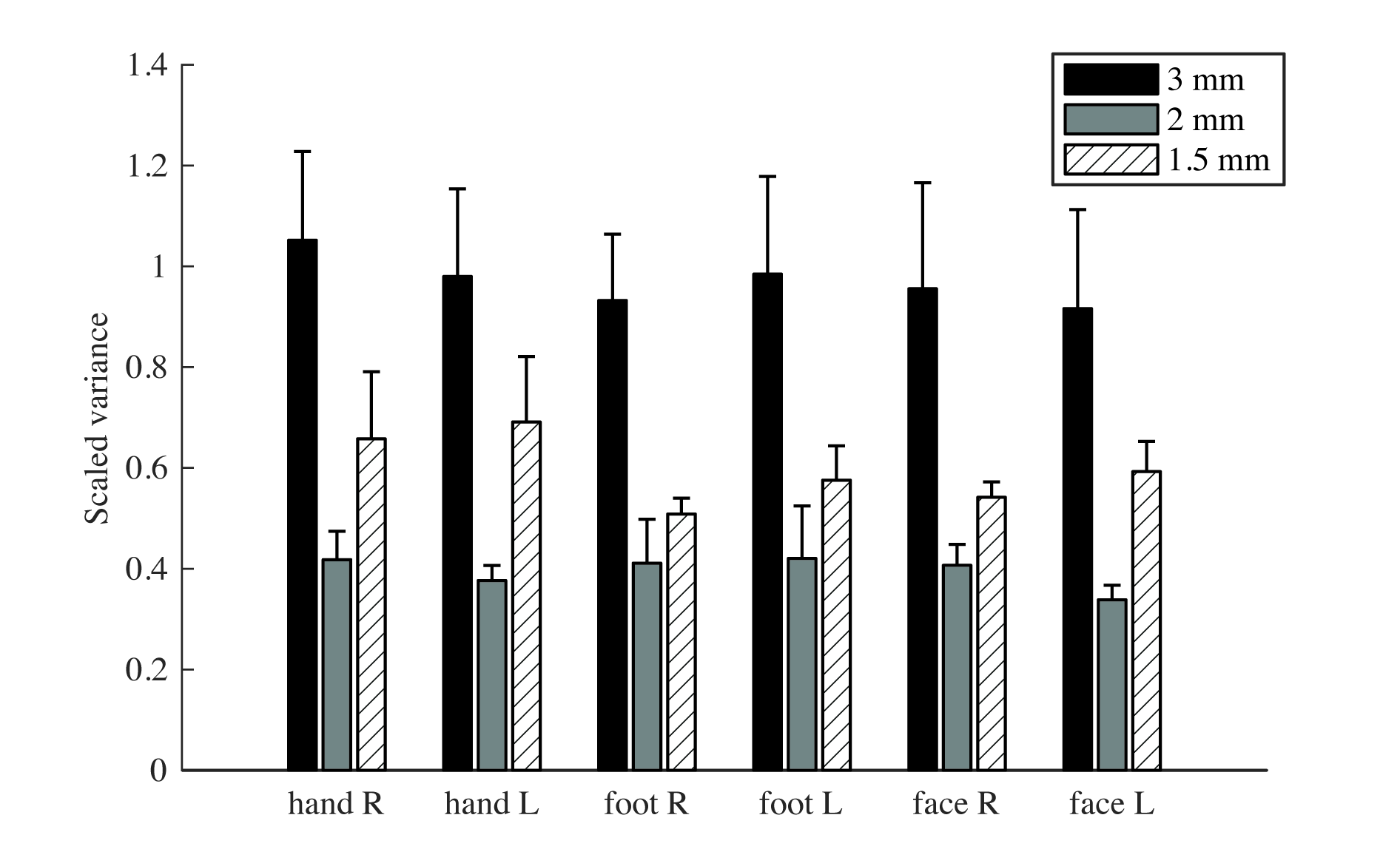

Supplement: FIGURE S5 — Average scaled signal variance of somatotopy maps across spatial resolutions. [file Image_5.TIF]

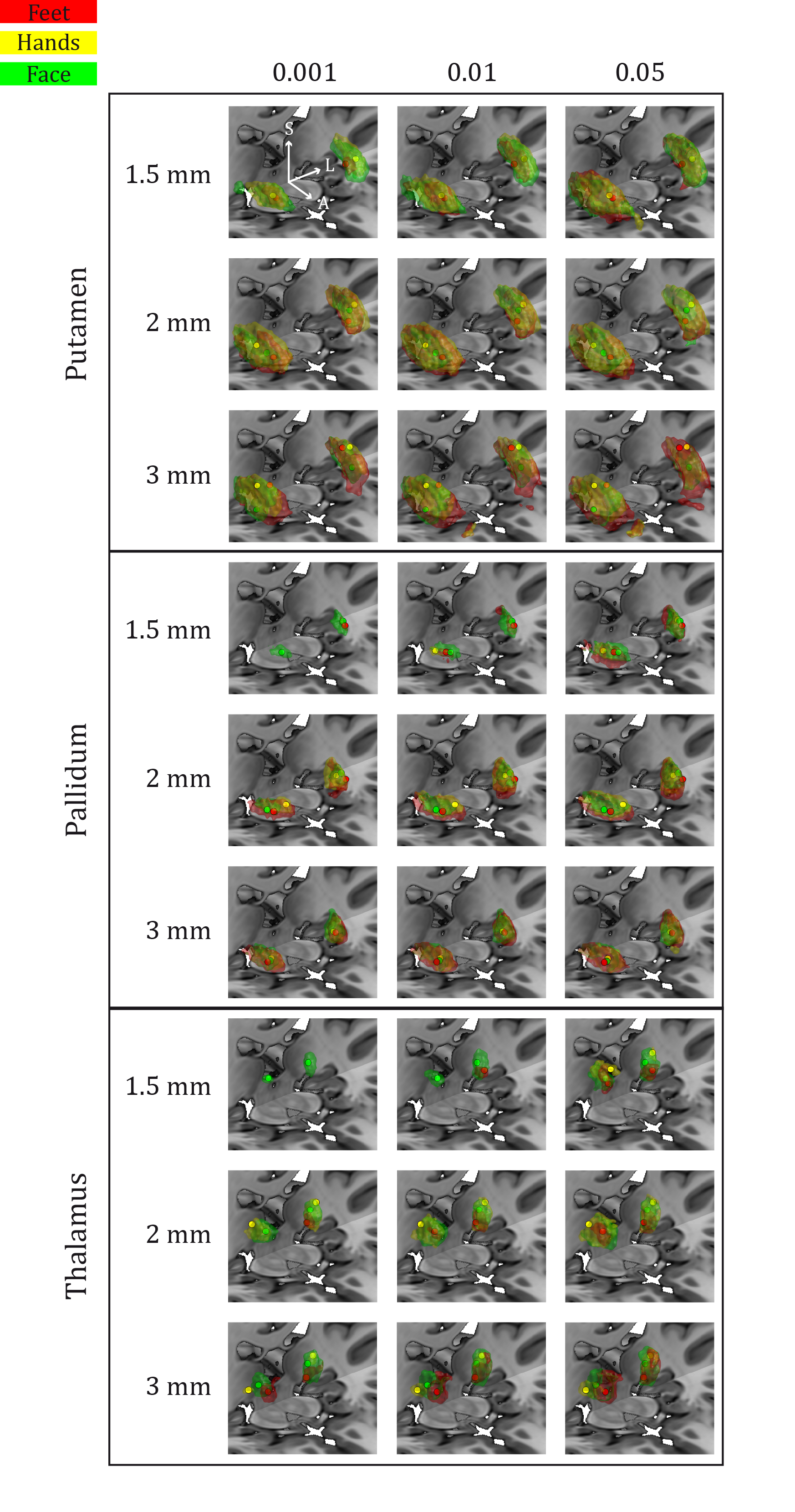

Supplement: FIGURE S6 — Surface rendering of motor somatotopy patterns in subcortical regions as a function of resolution and statistical threshold projected on canonical anatomical image in standard space. Group results from flexible factorial design represented as binarised statistical parametric maps after F-test across subcortical regions-of-interest, spatial resolution and statistical threshold (p < 0.001, 0.01, and 0.05, uncorrected for multiple comparisons, minimal cluster extent of 10 voxels). Filled circles indicate coordinate with maximal F-value for each contrast (red, feet; yellow, hands; green, face). White arrows in the top left image indicate directions of anatomical landmarks (S, superior; L, left; A, anterior). [file Image_6.TIF]

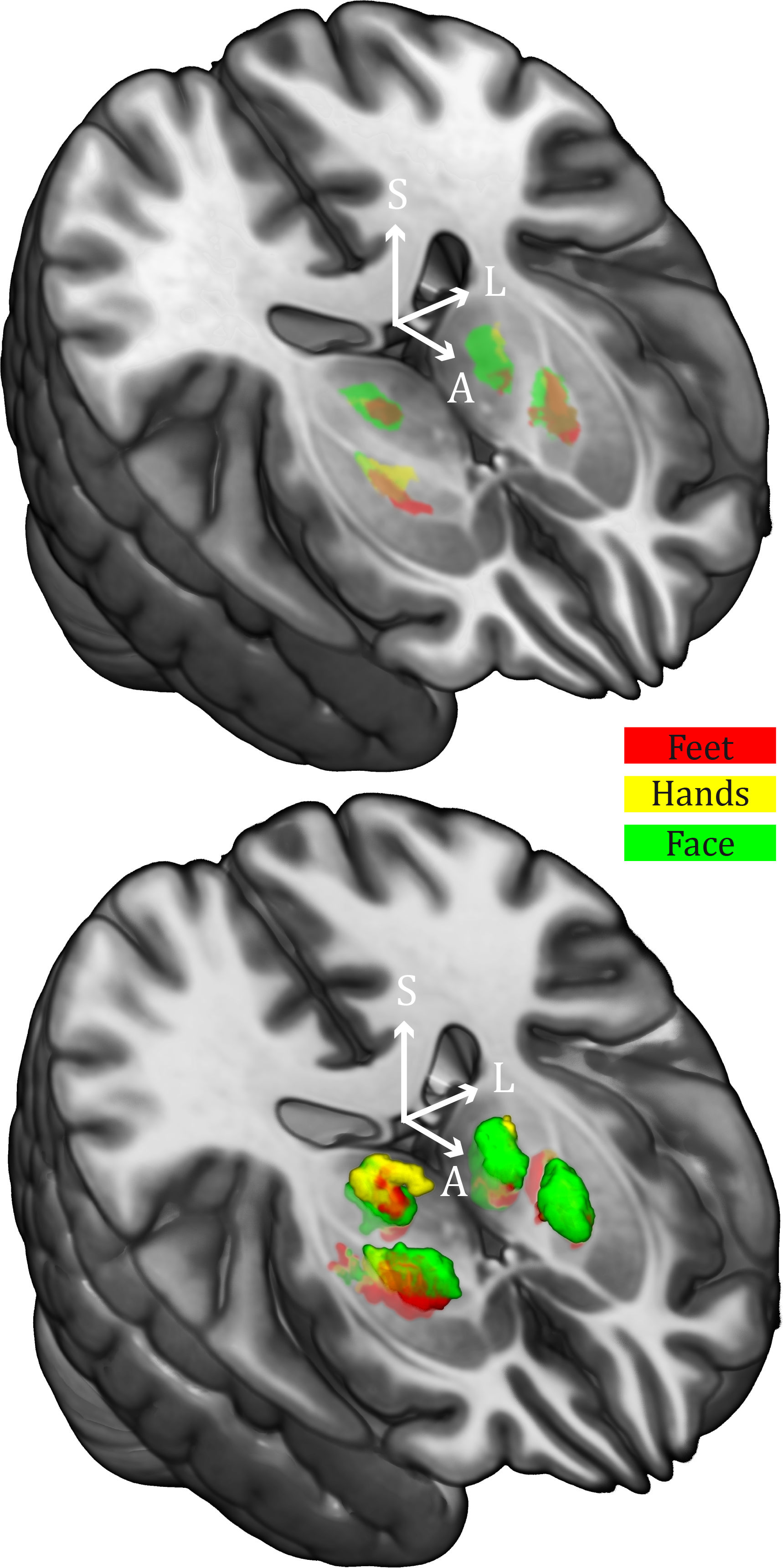

Supplement: FIGURE S7 — 2D and 3D representations of motor somatotopy at 1.5 mm resolution projected on canonical anatomical image in standard space. Non-thresholded statistical maps – F-contrasts for feet (red), hands (yellow) and face (green) at α = 0.01 uncorrected with a minimal cluster extent of 10 voxels. White arrows – directions for anatomical landmarks (S, superior; L, left; A, anterior). [file Image_7.TIF]

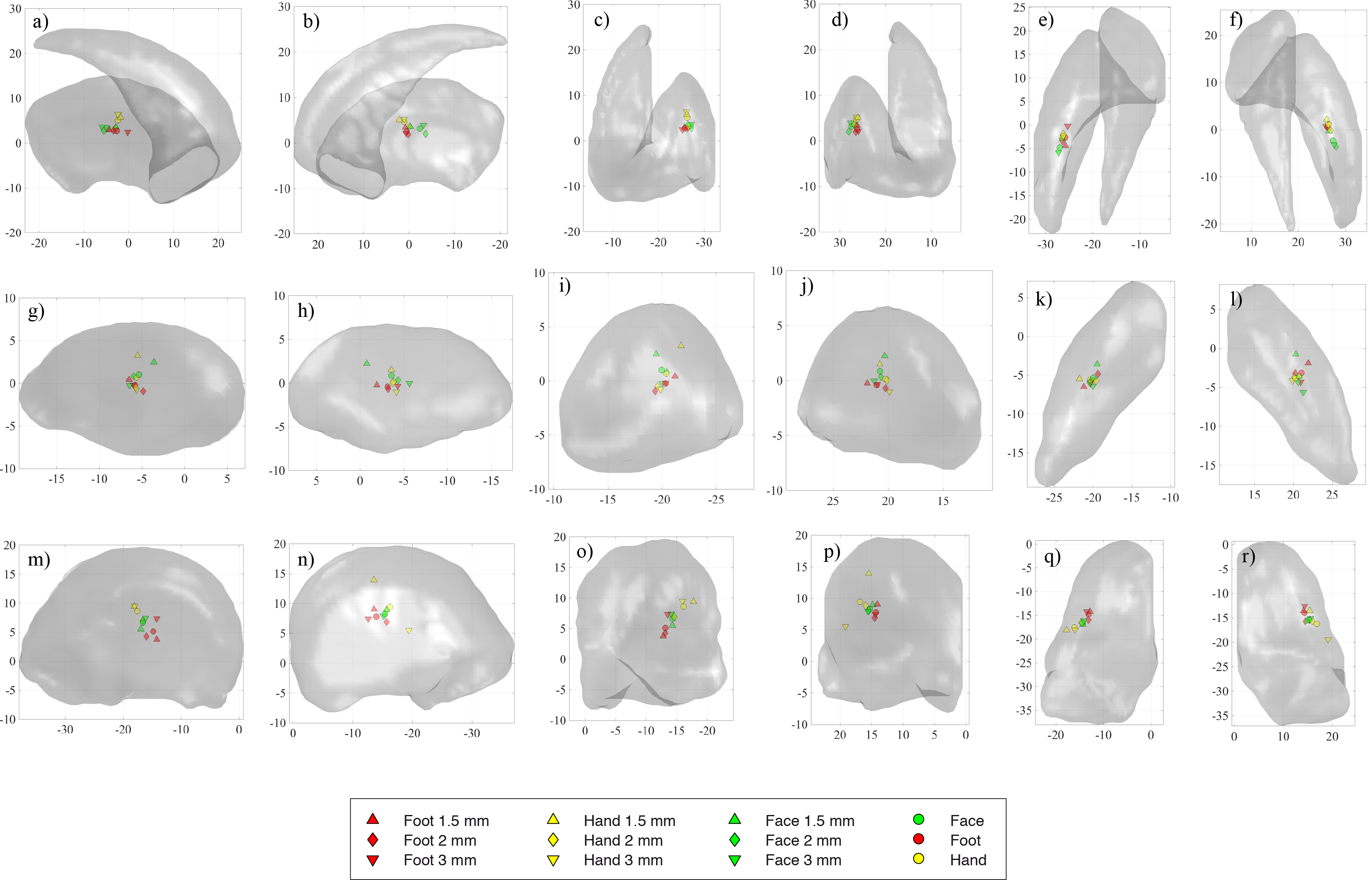

Supplement: FIGURE S8 — 2D projections of centers of mass coordinates for foot (red), hand (yellow) and face (green) movements at 1.5 (upward triangle), 2 (diamond) and 3 (downward triangle) mm resolution along X (a, b, g, h, m, and n), Y (c, d, i, j, o, and p) and Z (e, f, k, l, q, and r) planes in the putamen (upper row), pallidum (middle row), and thalamus (bottom row). Circles represent the average coordinates of centers of mass across resolution. [file Image_8.TIF]

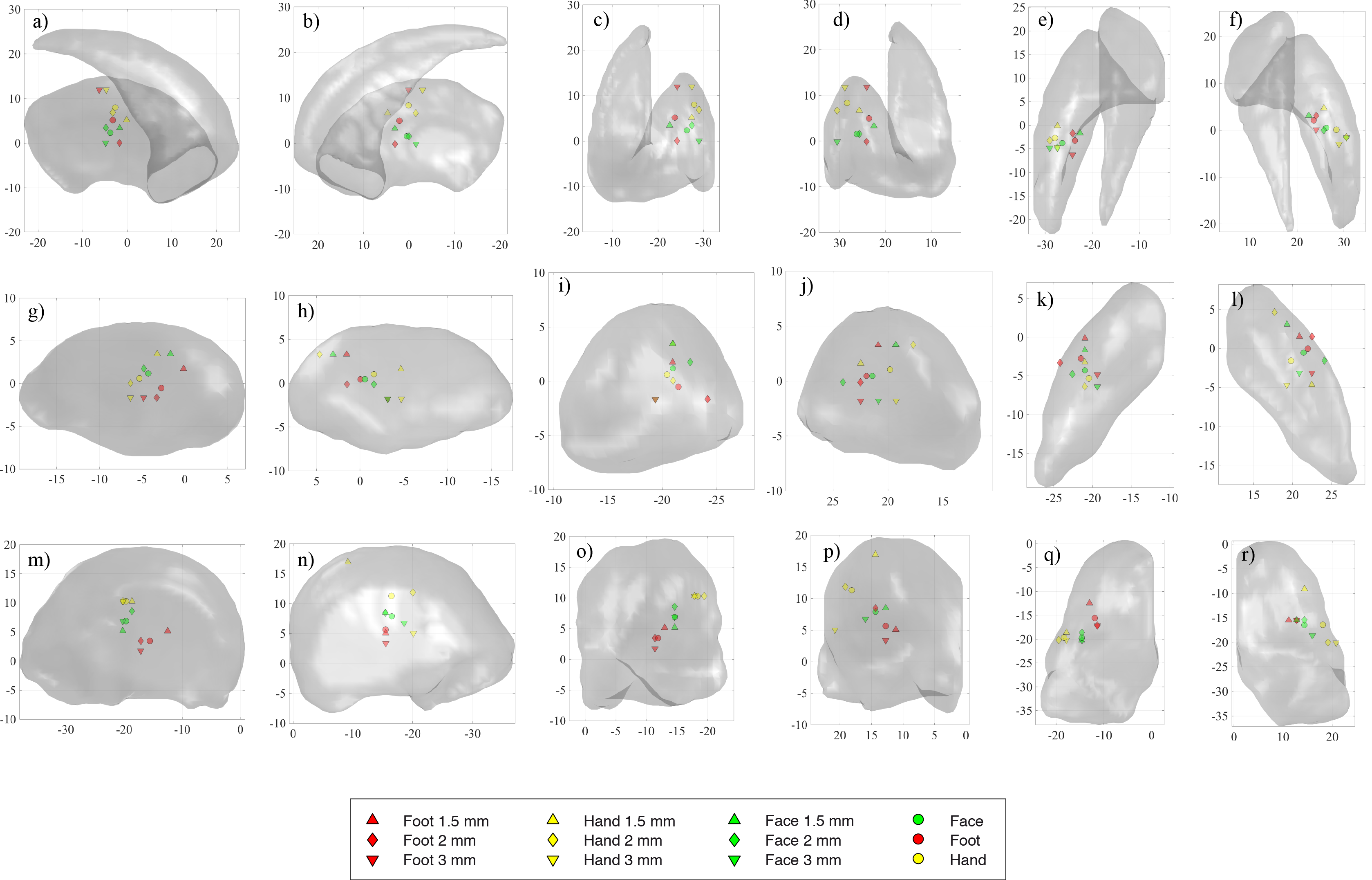

Supplement: FIGURE S9 — 2D projections of activation maxima coordinates for foot (red), hand (yellow) and face (green) movements at 1.5 (upward triangle), 2 (diamond) and 3 (downward triangle) mm resolution along X (a, b, g, h, m, and n), Y (c, d, i, j, o, and p) and Z (e, f, k, l, q, and r) planes in the putamen (upper row), pallidum (middle row), and thalamus (bottom row). Circles represent the average coordinates of activation maxima across resolution. [file Image_9.TIF]
